# From developing to developed: Mechanisms of health inequalities among seniors in China and Japan under macro-field control

**DOI:** 10.3389/fpsyg.2022.956165

**Published:** 2022-10-05

**Authors:** Ai-Bin, Lin Shengai

**Affiliations:** ^1^Department of Sociology, School of Ethnology and Sociology, Minzu University of China, Beijing, China; ^2^Department of Political Science, School of Management, Minzu University of China, Beijing, China

**Keywords:** health inequalities, field, developing countries, developed countries, social economic, health inequalities, population aging

## Abstract

The behavioral characteristics, health statuses, and survival times of seniors in China and Japan using the fixed cohort method and constructed relationship models among capital, habitus, and health based on Pierre Bourdieu’s social theory of practice. It was first found that capital, habitus, and health have a capital-based triangle generative structural relationship. Second, basic sources of health inequalities include the direct effect of capital and the indirect effect of capital through habitus, i.e., class habitus controlled by capital has class attributes and is also one of the sources of health inequalities. Third, time-space conversion of the field is not just the change in the total amount or composition of an individual’s capital but also includes the development and improvement of the macro-social environment, causing altered intensities of the impacts of capital and habitus on health. Fourth, the macro-social structures of developing countries significantly differ. The direct effect of capital on health is far greater than the indirect effect of capital on health through habitus, and health inequalities are mainly derived from the direct role of capital. Fifth, with socioeconomic development and improvements in social welfare systems, health inequalities have been generally reduced but have not been eliminated, and the mechanism of health inequalities in developed countries has gradually shifted from the direct effect of capital to class habitus.

## Introduction

It is generally accepted that health inequalities are systemic health differences brought about by social structural factors, while the health differences brought about by an individual’s initiative factors are not in the category of health inequalities. For example, it is suggested that health inequalities be studied through the social dimension, which includes social class, occupation, social hierarchy, gender, income, education, and culture, i.e., health differences caused by socioeconomic characteristics (Wagstaff and Van Doorslaer, 2000). Health inequalities are a manifestation of social inequality, the essence of which is the presence of systematic health differences among social groups with different advantages, for example, low-income groups, ethnic minorities, and women are more exposed to social inequalities such as health risks and illnesses than other social groups (Braveman, 2006).

However, social structural factors and individuals’ initiative factors do not exist in isolation. From the above viewpoint, the main motivation only takes the direct impact of structural factors on health into account and neglects the dependence of initiative factors on structural factors, i.e., it ignores the effects and limitations of structural factors on initiative factors. Therefore, health inequalities are not just the systematic health differences brought about directly by social structural factors but include the portion of health disparities brought about by social structural factors though affecting and limiting individuals’ initiatives. So, the structural relationships among three elements, i.e., structural factors, individuals’ initiative factors, and health practices at the micro-level and how the social development and advancement at the macro-level regulates the relationship among the three elements at the micro-level have become important issues for academia to solve.

**FIGURE 1:**
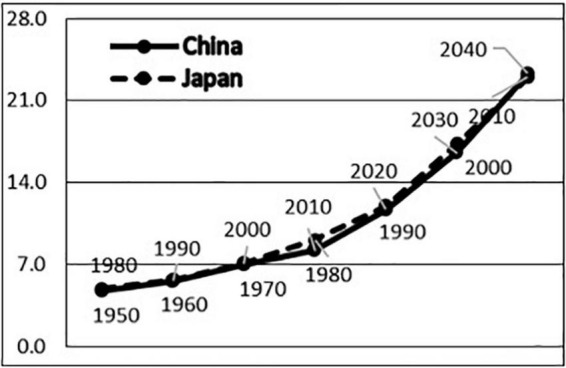
Comparison on population aging processes in China and Japan.

The proportion of seniors aged 65 or older in China was consistently less than 5.0% before 1980, began to rise sharply in the 1980s, reached 7.0% in 2000, and is expected to rise to 14.0% in 2025 (Ren and Hou, 2011). On the other hand, the proportion of elderly people aged 65 or older in Japan was lower than 5.0% before 1950, began to rise rapidly in the 1950s, and reached 7.1% in 1970 and 14.5% in 1995 (World Statistics, 2016). In terms of the number of years needed to double the aging rate, which reflects the aging speed of an aging population, despite the three-decade time lag in the aging process, the aging speeds of China and Japan are almost identical. The first phase is the rapid growth period, during which both countries took less than 20 years to increase the proportion of the elderly population from the long-term stable 5.0 to 7.0%. The second phase is the aging period, in which both countries took approximately 25 years to increase the proportion of the aging population from 7.0 to 14.0%. The third phase is the aged stage, in which both countries took approximately 15 years to increase the proportion of the aging population from the long-term stable 14.0 to 21.0%. Therefore, both countries have the characteristics of attaining population aging in a short time, which is different from the long process of population aging that has occurred in Europe (Figure 1).

If the social developments of China and Japan are adjusted by 30 years, the macro-society structures such as social issues, economic development, and welfare system in the three phases of population aging of the two countries have drastic similarities. In the first phase of rapid growth, both China (1980–2000) and Japan (1950–1970) entered an aging population society during the process of fast social transformation and rapid economic development. China experienced the end of the turmoil of the Cultural Revolution, the transformation from the planned economy to a market economy, and an economic growth rate of 5–13%, while Japan experienced the end of the war, the transformation from a controlled economy to a free economy, and an economic growth rate of 6–13%. The economic development of the two countries has significantly improved health problems centered on infectious diseases, malnutrition, and infant mortality, and average life expectancy has been greatly extended (Li, 2016). In the second phase of the aging period, both China (2001–2025) and Japan (1971–1995) were faced with the reality in which important factors affecting health have been shifted from social factors such as social structures and lifestyle to the stratification of social classes and the intensification of living pressure (Hideki, 2005; Kaori, 2007; Haruko, 2011), and lifestyle-related diseases, chronic diseases, and mental and psychological diseases have become the core of health problems. In the third stage of the aged period, both China (after 2025) and Japan (after 1995) are faced with the major social problems of social endowment and long-term care. At this stage, Japan accelerated the establishment of an endowment-related social security system, implemented the long-term care insurance system in 2000 based on a pension system and medical insurance system, and established a highly sophisticated social security system with the above-mentioned three major systems as the pillars (Zhao et al., 2014). It is projected that China will enter the aging society in 2025, will build a moderately prosperous society in all respects in 2030, and will face similar problems in establishing a more sophisticated social security system.

In summary, on the basis that China and Japan have high comparability concerning the process of population aging, social issues, economic development, welfare systems, and the respective coping strategies, in this study, we viewed China and Japan as comparable fields of developing and developed countries, respectively, to investigate and reveal the basic source of health inequalities, the main source of health inequalities in developing countries, the effects of macro-economic development and social welfare system improvement on the reduction of health inequalities, new mechanisms of health inequalities in developed countries, etc., to provide policy bases for countries at different stages of development in coping with population aging and continuously reducing the social inequality of the elderly.

## Literature review on studies of health inequalities of the elderly

### Source and formation mechanism of health inequalities of the elderly

The causal relationship between structural issues such as socioeconomic status and health is controversial (Warren, 2009), of which two theories are present: the social causality theory and the health choice theory (Elstad and Krokstad, 2003). Fuqin (2012) argued that health inequalities are the reproduction of inequalities of social class and status, and in the context of current China, the social causality theory has higher explanatory power than the social choice theory. Sociology mainly discusses how socioeconomic status inequalities generated by social structures lead to health inequalities (Robert and House, 2000). Health conditions are controlled by social structural factors, i.e., the status of an individual in social structures determines the individual’s health level, and a positive correlation between the individual’s socioeconomic status and the individual’s health status is present (the lower the socioeconomic status, the lower the health status) (Feinstein, 1993; Dahl, 1996; Mackenbach et al., 1997). As a developing country, China also has health inequalities brought about by social structural factors such as the social welfare system and the individual’s socioeconomic status. With aging, the physical functions of the seniors from different socioeconomic status groups differentiate continuously (Jiao, 2014); the higher the socioeconomic status of the elderly, the better their health status (Ma and McGhee, 2013), and the longer their life expectancy and healthy life expectancy (Tang et al., 2004). Seniors with different types of social and medical insurance policies show significant differences in health (Huang and Wu, 2010; Liu, 2014), and having a pension or not having a pension has a significant impact on the risk of death among the elderly (Zeng, 2007).

The state of macro-social development in each country or region regulates the relationships between social structures and health inequalities (Sheng et al., 2022). The relationship between socioeconomic status and health is constrained by macro-social structural factors such as the social, political, and economic conditions of a country or a region (Lowry and Xie, 2009); in developed countries, socioeconomic status has a stronger impact on health (Wilkinson, 1997), while in developing countries such as Latin America and the Caribbean countries, the relationship between health and socioeconomic status is not so profound (Dachs et al., 2002). Chinese scholars have found that improvements in the level of urban public health services weaken the negative effects of rising income inequality on health inequalities (Huang, 2012), and factors such as major cities can alleviate the level of the income difference-based health inequalities (Deng, 2010); with the economic development and the establishment of the social security system, health inequalities among the elderly are becoming more severe (Du and Wang, 2013). Therefore, the degree of the relationship between socioeconomic status and health will be regulated by macro socioeconomic development (Bassu et al., 2002; Sathesh et al., 2020).

Formation mechanism of health inequality (Zheng and Yin, 2022): Many studies have demonstrated that social structural factors exert an impact on health but have rarely elucidated the theoretical mechanism of how socioeconomic status affects health (Mirowsky et al., 2000). According to the social causality theory, the influencing mechanism can be categorized based on two aspects (Wu and Zhu, 2021). The first is the material environment theory, which claims that the income and living environment gaps brought about by socioeconomic status are important factors affecting health; the second is the lifestyle theory (Chen et al., 2021), which argues that different lifestyles brought about by socioeconomic status are an important factor affecting health (Zheng et al., 2022a). The better health enjoyed by people with higher socioeconomic status is because they have a good work and living environment, which lowers their chances of getting hurt (Evans and Kantrowitz, 2002); moreover, they are in an advantageous position in terms of accessibility and utilization of healthcare resources and services (Victora et al., 2000; Meng, 2007; Xie, 2009; Maheswaran et al., 2017). However, Gu and Zeng (2004) argued that socioeconomic status only has a limited impact on the physical health of the elderly such as their ability to care for themselves. Therefore, the material environment theory should only be applied to the development stage at which a large social gap is present. Wang (2012) validated the lifestyle theory from the perspective of sports and medicine and argued that the higher the socioeconomic status an individual has, the more likely the individual will have and maintain a healthy lifestyle, which in turn has a direct impact on the individual’s health (Zheng et al., 2022b).

### Unsolved problems from previous studies

First, previous studies were generally performed from the perspective of the contradiction between social structures and an individual’s initiatives and lacked a research paradigm that transcends the contradiction and treats the two as a combined action (Choi et al., 2021). Moreover, they only paid attention to the health differences brought about directly by social structural factors but ignored health inequalities caused by the dependence of the individual’s initiative factors on social structural factors.

Second, previous studies lacked comparative investigations of the sources of health inequalities at different macro development stages and concluded that the more developed a society is, the greater the health differences by simply using macro data, leading to the dissociation between micro-analysis and macro-analysis. Some studies attributed the intensification of health inequalities with socioeconomic and social security development to the unfair distribution of social material resources and ignored the limitations of the role of physical resource inequalities in health inequalities.

Third, previous studies lacked complete health indicators, were unable to portray health simultaneously from the aspects of health status and survival time, and were especially inadequate in portraying the health of the elderly with indicators of self-care ability. Most studies only used cross-sectional survey data to examine the relationships between social structural factors and health inequalities and between an individual’s behavioral factors and health status. Although some studies did conduct follow-up surveys, they only tracked life and death events rather than survival length.

Fourth, the statistical analysis methods adopted by previous studies are not suited to examine the multiple direct and indirect effects between abstract concepts, resulting in the separation of theoretical model discussion and data model analysis. Some studies used different models to separately examine the relationships between socioeconomic status and health and between lifestyle and health but subjectively inferred that socioeconomic status influences health through lifestyle changes.

## Theoretical perspective and hypotheses of this study

To overcome the contradiction between social structure and an individual’s initiatives, the separation of micro-analysis and macro-analysis, and the dissociation between theoretical models and empirical data in previous studies, in this section, based on Bourdieu’s social theory of practice, we first establish the theoretical basis for the formation of a health field with the capital that reflects the individual’s position in social structures, the habitus that reflects the individual’s initiatives, and the macro-social environment, present hypotheses on generating relationships among three concepts (capital, habitus, and health) on the basis of theoretical revision, and finally apply the hypothesis models to different health fields of China and Japan with different stages of social development to examine the differences in the relationships among the three concepts at different stages of social development and reveal the characteristics and mechanism of health inequalities occurring in both developing and developed countries.

### Theoretical construction of this study

**FIGURE 2:**
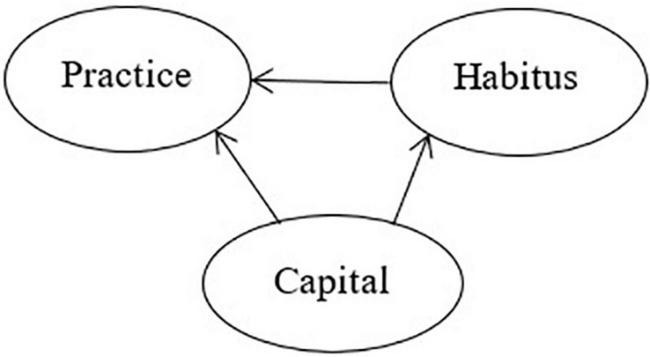
Triangular generative relationships among capital, habitus, and practice.

First, Bourdieu’s social theory of practice introduces capital and habitus, two basic concepts that lay the foundation for transcending the contradiction between social structures and individuals’ initiatives and the discussion on generative structural relationships among capital, habitus, and practice. The social practice theory emphasizes the primacy of relations. First, various objective structures (various location spaces, i.e., capital), i.e., the distribution of socially effective resources, are constructed; it is the state of these socially effective resources that determines the external constraints on interactions and appearances. Then, the actor’s direct experience is introduced to reveal various perceptions and evaluations (various dispositional tendencies, i.e., habitus) of the actions that are constructed from within. Although the above two links are indispensable, they are not completely equivalent; because the actor’s viewpoints will fundamentally change with the different positions the actor occupies in the objective social space, the objectivist observation epistemologically precedes the subjectivist understanding. Moreover, the corresponding relationship of domination and being dominated as well as the generative relationships are present between social structures and mental structures, which further extend to the analysis of subjective dispositional tendency and thus ease the contradiction between subjectivity and objectivity (Bourdieu, 2015, p. 10–14). Bourdieu further interpreted the relationships among objective structure, habitus, and practice as the linear generative relationship of structure-habitus-practice (Bourdieu, 2015, p. 269), in which the relationships among capital, habitus, and practice not only are one-to-one correspondences but also consider that some habitus is the generative result of capital. Further, Bourdieu metaphorized habitus with “calligraphy” to describe principled attributes of habitus; although an individual’s handwriting is related to writing paper or blackboards and pen or chalk, the individual’s calligraphic style is always similar and recognizable (Bourdieu, 2015, p. 272). However, if we expand the scope of observation to include “whether” there is writing paper or blackboards and pen or chalk, as well as “whether the individual is capable of” writing as the selections of the formation condition of handwriting, we will find that the practice of handwriting is the first subject to the direct influence of objective conditions and then to the indirect effect of objective conditions through the writing style. In other words, capital not only establishes a generative relationship with practice through habitus but also directly plays a generative role in practice, i.e., the three form a capital-based triangular generative structure. The health practice has generative relationships with the above two elements but is not entirely dependent on capital, and in the theoretical model, other unknown confounding factors are also present, giving rise to the theoretical model of micro-analysis transcending the contradiction between social structures and individuals’ initiatives (Figure 2).

Second, the concept of habitus proposed by Bourdieu provides a new perspective and operational foundation for the study of initiatives. Bourdieu’s social philosophy strives to seek and grasp the intention without intentionality and the knowledge without cognitive purpose and to acquire the ability to grasp the pre-reflection subconsciousness on the social world generated through the actor’s long-term immersion in the social world. Thus, Bourdieu thinks habitus is creative and reflects the imagination but is confined by its structures, which are deposits of social structures at the body level (Bourdieu, 2015, p. 18–19). Reasonable behaviors in a medical sense such as eating, sleeping, smoking, drinking, and walking have strong instrumental rationality and passiveness, purporting at the expected instantaneity of health returns rather than reflecting the individual’s habitus of subjective initiatives. Habitus is the “collective unconsciousness” of those who occupy the same position and provides cognitive and emotional guidance, enabling individuals to depict this world in a collective way and to sort, select, evaluate, and act with a unique attitude (Jonathan, 2006, p. 472). Therefore, habitus should be the embodied subjective initiatives, a pursuit for the meaning of life or meaning of living, with autonomous and cultural ideals characterized by exploration, taste, and links.

Moreover, habitus is the collective individualism achieved through embodiment or the collectivization of biological individuals attained through socialization (Bourdieu, 2015, p. 19). Although comprehensive qualities such as taste created by habitus seem to be simple, natural, and personal phenomena, they are covariant with the objective positions of class, reflecting the cultural hierarchy, and thus, the social hierarchy of objective positions of class, and the conflicts are all class ones (Jonathan, 2006, p. 472). Habitus can be decomposed into class habitus and personal habitus, the former being class attributes formed through the restriction and influence of capital, and the latter being the individual’s characteristics formed through the influence of other factors. Class habitus is the internalized form of class conditions and is affected by class conditions. Therefore, class habitus, which is derived from capital, is one of the sources of health inequalities.

Third, Bourdieu’s theory of social practice introduces the concept of field, which lays the foundation for transcending the contradiction between macro-analysis and micro-analysis and examining the effect of capital and habitus on health practice at different stages of macro-social development. Bourdieu believes that a field is composed of a series of objective historical relationships among various positions attached to a certain form of power (or capital), a system in which various objective forces are adjusted and finalized, and a relationship configuration that is endowed with a given gravity that is imposed on the objects and actors that have already entered the field (Bourdieu, 2015, p. 15–16). In this study, we decompose social structures into the macro-level social institutions and the micro-level individual’s social positions, the former forming various gravitational beams of a given field to control the interactions among major factors of the microscopic society. On the one hand, the direct and indirect effects of the amount of capital brought by the individual’s social position on health practice (Heydarpour et al., 2020) are analyzed from the micro perspective, and on the other hand, from the macro perspective, the field gravitation formed by macro-social development such as social welfare system, population structure, interest stratum, and group exists external to individuals but regulates the intensity and method of capital’s impact on health practice. At the same time, Bourdieu’s space-time conversion theory addresses the changes in the distributions and relative proportions of various capital forms, which are equivalent to the change of the structure of the field, so that the field acquires a certain historical dynamic changing and calibrating capability, and the determinism of the traditional deconstructionism that totally lacks elasticity for alternatives is thus avoided (Bourdieu, 2015, p. 17). The spatio-temporal field shift in this study is not just the changes in the total amount and structure of the individual’s capital, but, more importantly, with social development and sophistication, the changes in the gravitational forces formed by a variety of external macro-social structures in a health field (Malekshah et al., 2022) which prompted the changes in the manner and intensity of the action of the individual’s capital on health practice (Malekshah and Ansari, 2021).

In the field of developing countries where the macro-society is in its initial development stage and the social welfare system is not yet sophisticated, the field gravity formed by the low coverage of the welfare system such as the medical insurance system as well as the low reimbursement rate strongly amplify the impact of medical resources and food resources brought about by the individual’s social position on health, whereas habitus such as subjective dispositional tendency has little effect on health. In the field of developed countries where the macro-society is highly developed and the social welfare system is highly sophisticated, the equalizing forces such as the macro-social medical resources and food resources prompt a significant decrease in the direct effect of the individual’s capital on health; relative to medical and food resources, habitus, which is difficult for the macro-social system to intervene, will exert a significantly increased influence on health.

### Theoretical hypotheses of this study

**FIGURE 3:**
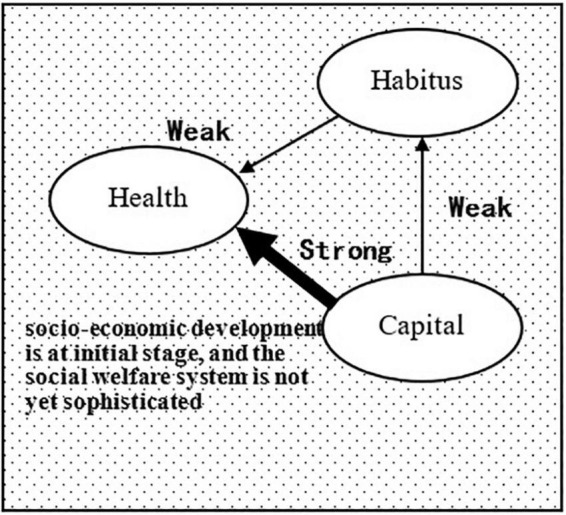
Health field of the elderly in developing countries.

Hypothesis 1: The individual’s capital and habitus simultaneously affect health, and the capital is in a dominant position and also affects habitus, giving rise to a triangular generative structural relationship. Habitus can be decomposed into class habitus (affected by capital) and personal habitus (affected by other factors). Therefore, health inequalities have two sources, one of which is the direct effect of capital on health and the other of which is the indirect effect of capital on health through class habitus. In other words, class habitus has a class nature, which is also one of the sources of health inequalities.
Hypothesis 2: The time-space conversion of the health field is not just changing in the total amount of capital and the capital composition at the micro-level over time but includes changes in field gravitational forces formed through the advancement of socio-economy and the sophistication of the social welfare system at the macro-level, which will lead to changes in the intensity of the effect of capital in the micro-analysis and habitus on health.
Hypothesis 3: In the health field of the elderly in developing countries where the macro-social-economic is in its initial development stage and the social welfare system is not yet sophisticated, the direct effect of capital on health is much greater than its indirect effect on health through habitus. The capital-dependent class habitus only has a very weak effect on health, and the source of health inequalities is mainly the direct effect of capital (Figure 3).

**FIGURE 4:**
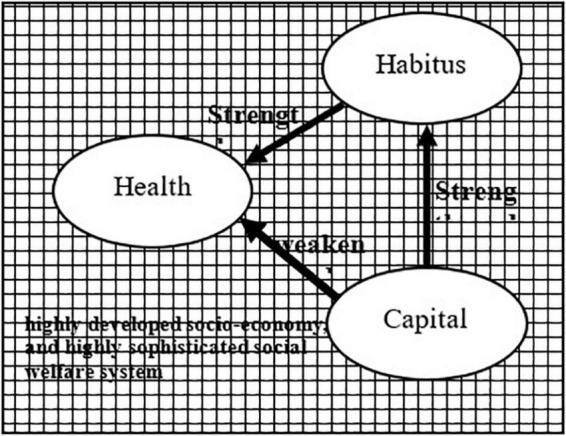
Health field of the elderly in developed countries.

Hypothesis 4: In the health field of the elderly in developed countries where the macro-society is highly developed and the social welfare system is highly sophisticated, in general, health inequalities have been reduced but have not yet been eliminated, and the direct effect of capital on health has decreased while the indirect effect of capital through habitus has increased; class habitus has gradually become an important source of the generation of health inequalities (Figure 4).

## Research method

### Research approaches

First, three concepts at the micro-level, i.e., capital, habitus, and health, as well as their operational indicators were established. The triangular generative structure model was then constructed based on the revision theory, and the relevant data of various controllable exposure factors, behavioral characteristics, health statuses, and survival times that are included in the concepts of capital and habitus of seniors in China and Japan, were collected using the fixed-cohort study method.

Then, China and Japan were compared and analyzed as the references for the health fields of developing countries and developed countries, respectively. The bases for the comparison between China’s Shenyang City and Japan’s Tama City are that both cities belong to large metropolitan areas dominated by secondary and tertiary industries. The subjects were the seniors over the age of 60 that are retirees mainly staying at home, with pensions as their main income source, and vastly different macro-social environments such as social welfare systems, aging, and living standards.

### Survey methods in Shenyang city, Liaoning province, China, and Tama city, Tokyo, Japan

The survey in Shenyang City, China, was jointly executed in June 2000 by the Institute of Urban Science of the Tokyo Metropolitan University and the Health Bureau of the Shenyang Municipal Government in the Heping, Huanggu, Shenhe, Dadong, and Tiexi Districts of Shenyang City. From 450,000 seniors aged 60 and above, 4,460 from 10 neighborhoods in 10 sub-districts were sampled. The survey was performed by doctors from the Centers for Disease Control and Prevention (CDC) and staff from the community offices of each city district, and 3,654 questionnaires were recovered for a recovery rate of 81.9%. In 2012, baseline respondents were conducted with the fixed-cohort conversion survey, in which the respondents that were untraceable due to missing household registration information and address or due to being recently deceased after the last survey were excluded. The observation period was 2,920 days, from 1 January 2001 to 31 December 2008, during which 2,741 seniors were followed up, of which 506 were deceased during the period.

The survey in Tama City, Japan, was jointly performed in April 2004 by the Institute of Urban Science of the Tokyo Metropolitan University and the Elderly Welfare Bureau of Tama City on 16,462 seniors aged 60 and above through a questionnaire survey *via* mail, from which 13,067 questionnaires were recovered at a recovery rate of 79.4%. In 2010, the baseline survey respondents were followed up with the fixed-cohort death outcome survey, in which the respondents who were untraceable or recently deceased after the last survey were excluded. The follow-up period was 1,065 days, from 1 September 2004 to 31 August 2007, during which 8,282 seniors were followed up, of which 569 were deceased during the period.

### Operational indicators of analytical concepts (capital, habitus, and health)

Operational indicators of the concept of capital: Bourdieu’s concept of capital includes four types, i.e., economic capital, social capital, cultural capital, and symbolic capital (Jonathan, 2006, p. 468–469). In this study, the economic capital mainly used the measurements of economic income, in which the incomes of the subjects from China and Japan were categorized into three levels (high, medium, and low), which were valuated at 3, 2, and 1, respectively. Cultural capital was measured by education level, which was categorized into four levels (above junior college level = 4; high school or junior high school level = 3; elementary school level = 2; no education at all = 1). Because the subjects were all retired seniors, social capital and symbolic capital were not used in this study.

Operational indicators of the concept of habitus: Habitus is the actor’s disposition system, not only guiding the distinction principle of social distinction but also being the distinction activities that actually play the differentiating role. Personal lifestyle and hobbies are important manifestations of habitus and allow individuals to unconsciously engage in various modes of social activities; they can objectively be adapted to their purposes without having to set conscious goals for those purposes nor the specific control on the procedures that must be taken to achieve such goals (Gao, 2017, p. 833–847). Therefore, habitus behavior itself has the nature of interest and taste rather than medically sound work-rest rules and passive instrumental behaviors. In this study, habitus is operationally divided into two tendencies, group interest, and personal interest, for which operational variables are collective community activities such as participating in volunteer activities, and community activities as well as personal entertainment interests such as calligraphy, games, and hobbies.

Operational indicators of the concept of health: Two aspects, i.e., health status and survival time, were considered in this regard. The baseline survey used a health assessment scale that measures 14 types of disease, eight types of discomfort symptoms, and 13 types of life skills. With respect to physical health, five items, i.e., suffering from diseases such as cerebrovascular diseases and other diseases, discomfort symptoms such as waist and leg pain, life skills such as making deposits and withdrawals at a bank, and paying utility bills, were examined in this study. Social health refers to the individual’s interactions with others and the social environment, good interpersonal relationships, and level of playing social roles, including two aspects, i.e., human interaction and spatial interaction. In this study, the frequency of outing activities and the frequency of interaction with friends and relatives were considered. Mental health refers to an individual’s subjective feelings on the body and emotions, including two aspects, i.e., feelings regarding the body and feelings regarding life. In this study, two items, i.e., subjective health perception and life satisfaction, were measured. The survival time of the concept of health is an important feature of this study and was measured by the number of days the respondent survived from the date of the baseline survey to the end of the follow-up survey; the survival time was actually calculated a few months after the baseline survey started to reduce the impact of recent deaths after the baseline survey.

### Analysis method

The choice of analysis subjects: The health indicators used in this study included survival time. Some of the subjects had deceased at the end of the follow-up survey, and in that case, if all the subjects that were followed up were used as the analysis subjects, it would lead to the survival of the majority being a constant, thus masking the survival difference derived from various factors. Therefore, in this study, the subjects from the two cities who were deceased during the follow-up survey were used as the analysis subjects so the effects of capital and habitus on the overall health inequalities could be further clarified.

Choice of statistical methods: In this study, we used the structural equation model to perform statistical analyses, which combines several multivariate statistical techniques such as factor analysis, path analysis, and regression analysis, and were able to resolve multiple causal relationships as well as direct and indirect causal relationships. In particular, it uses latent variable techniques to enable theoretical concepts to be operational and is thus a statistical analysis technique that is closest to theoretical analysis. Because health indicators describe two aspects of health, i.e., “health status” and “survival time,” the theoretical hypotheses of the study were translated into the relationships among four variables, i.e., “capital,” “habitus,” “healthy status,” and “survival time.”

Choice of adjustment variable: Because the age factor may cause differences in the capital, habitus, health status, and survival time (Table 1), it was used as an adjustment variable in the model, and it was found that age only affected capital structure and health status and exerted no significant impact on habitus and survival time. For the sake of clarity of model relationships, the arrow lines of age on habitus and survival time were eliminated.

**TABLE 1:** Characteristics of the distributions of objects in Shenyang city and Tama city, Japan.

Concept classification				Operational variable	Valuation	Choices	Shenyang city, China (*n* = 506)		Tama city, Japan (*n* = 569)	
Attribute				Gender	1	Male	260	51.4%	325	57.1%
					0	Female	246	48.6%	244	42.9%
				Age group	1	Sixties	177	35.0%	36	6.3%
					2	Seventies	232	45.8%	232	40.8%
					3	Eighties and above	97	19.2%	301	52.9%
Capital			Economical	Income group	1	<500 Yuan	219	43.3%	179	31.5%
					2	500-999 yuan	248	49.0%	343	60.3%
					3	1,000 yuan and above	39	7.7%	47	8.3%
			Cultural	Education level	1	No educational at all	182	36.0%	1	0.2%
					2	Elementary school	222	43.9%	131	23.0%
					3	Secondary and high schools	90	17.8%	324	56.9%
					4	Vocational college, college and above	12	2.4%	113	19.9%
Habitus			Group interest	Community activities	1	Do not participate	328	64.8%	429	75.4%
					2	Occasionally participates	127	25.1%	96	16.9%
					3	Frequently participate	51	10.1%	44	7.7%
			Personal interest	Hobby	1	None	405	80.0%	271	47.6%
					2	One	79	15.6%	199	35.0%
					3	Many	22	4.3%	99	17.4%
Health	Health status	Mental health	Life satisfaction	Life satisfaction	1	Not satisfied	59	11.7%	119	20.9%
					2	Acceptable	83	16.4%	207	36.4%
					3	Very satisfied	364	71.9%	243	42.7%
			Health satisfaction	Self-assessment on personal health	1	Not healthy	87	17.2%	159	27.9%
					2	Acceptable	152	30.0%	118	20.7%
					3	Healthy	245	48.4%	233	40.9%
					4	Very healthy	22	4.3%	59	10.4%
		Social health	Spatial interaction	Outing frequency	1	Not at all	109	21.5%	110	19.3%
					2	2–4 times per month	55	10.9%	195	34.3%
					3	3–5 times per week	98	19.4%	131	23.0%
					4	Almost everyday	244	48.2%	133	23.4%
			Human interaction	Communication frequency	1	Not at all	74	14.6%	184	32.3%
					2	2–4 times per month	84	16.6%	299	52.5%
					3	3–5 times per week	130	25.7%	48	8.4%
					4	Almost everyday	218	43.1%	38	6.7%
		Physical health	Survival skills	Making bank visits	1	Capable	224	44.3%	344	60.5%
					0	Not capable	282	55.7%	225	39.5%
				Paying utility bills	1	Capable	247	48.8%	376	66.1%
					0	Not capable	259	51.2%	193	33.9%
			Disease type	Cerebrovascular disease	1	Yes	78	15.4%	72	12.7%
					0	No	428	84.6%	497	87.3%
				Other diseases	1	Yes	71	14.0%	86	15.1%
					0	No	435	86.0%	483	84.9%
			Discomfort symptoms	Waist and leg pains	1	Yes	135	26.7%	158	27.8%
					0	No	371	73.3%	411	72.2%
	Longevity	Time								
			Average number of days				1,595			567
			Distribution range				15–2,921		5–1,057	

The income levels in the case of Tama City are based on annual income, which are (1): below 2 million yen; (2): 2–4.99 million yen; and (3): 5 million yen and above. The tracking time in the case of Shenyang City is from Jan. 1, 2001, to Dec. 31, 2008, and that in the case of Tama City is from Sept. 1, 2004, to Aug. 31, 2007.

The statistical analysis software used in this study were SPSS18.0 and Amos17.0.

## Analysis results

### Evaluation of effectiveness of structural equation models for capital, habitus, and health

In the structural equation models, latent factors such as “health status,” “capital,” and “habitus” are represented by oval shapes. The observed variables such as “survival time” are represented by rectangular shapes, and the unknown factors of each of the variables are represented by z, d, and e, respectively, and the numbers above the arrow lines are normalized path coefficients that range from −1 to 1 and that represent the strength and direction of the effect. The goodness-of-fit index (GFI), which indicates the degree of fit between the constructed theoretical model and real data, was used to evaluate the validity of the model; it varies between 0 and 1, and the greater the value, the higher the level of fit. In academics, it is generally accepted that a GFI value above 0.9 indicates an ideal model, and the adjusted goodness-of-fit (AGFI) can avoid the false high value of a fit derived from too many connecting lines. Meanwhile, the lower the root mean square error of approximation (RMSEA) in the factor analysis, the better. In this study, the GFI, AGFI, and RMSEA of the model for Shenyang City were 0.640, 0.912, and 0.061, respectively, and those of the model for Tama City were 0.940, 0.912, and 0.064, respectively, indicating that the models had high validity.

**FIGURE 5:**
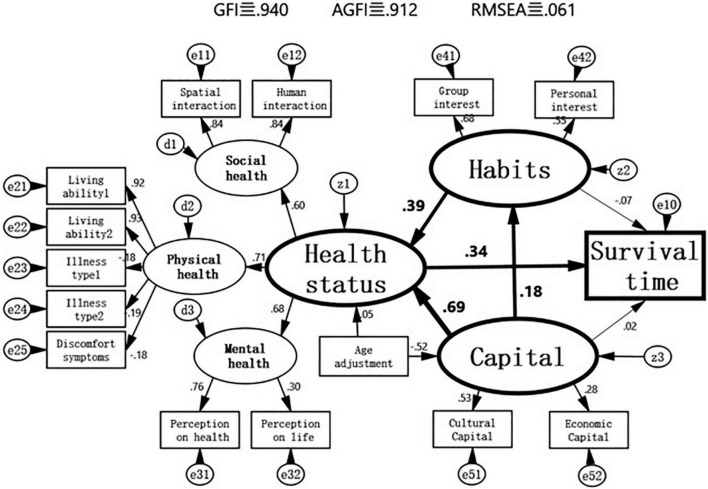
Structural equation model of capital, habitus and health of the elderly in Shenyang City, China.

**FIGURE 6:**
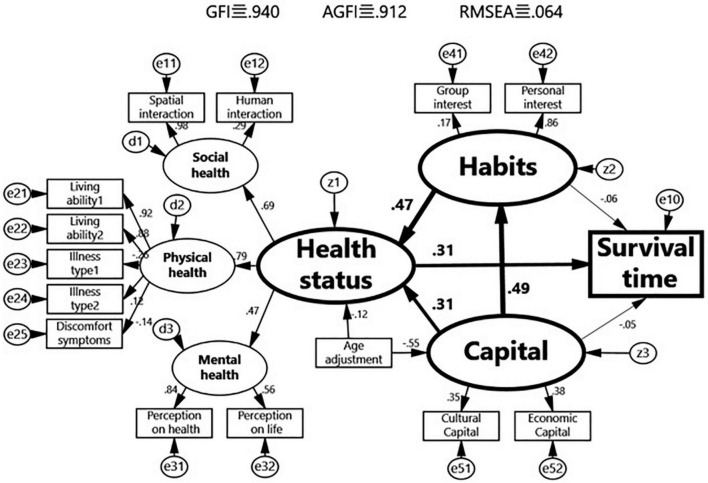
Structural equation model of capital, habitus and health of the elderly in Tama City, Japan.

The non-standardized path coefficients and test results between the latent factors and observed variables in the structural equations are shown in Table 2. The non-standardized path coefficients of “capital” on “health status” and “habitus,” the path coefficient of “habitus” on “health status,” and the path coefficient of “health status” on “survival time” all passed the significance test, whereas the non-standardized path coefficients of “capital” and “habitus” on “survival time” did not pass the significance test, indicating that “capital” and “habitus” exert no direct influence on “survival time” and only have an indirect influence through “health status.” The non-standardized path coefficient of “age” on “health status” did not pass the significance test, but that of “age” on “capital” passed. The factor analyses at other levels all passed the significance test, indicating that the operationalization of the latent concepts is reasonable (see Table 2 and Figures 5, 6).

**TABLE 2:** Non-standardized path coefficients and tests of structural equations of capital, habitus, and health.

			Shenyang city, China				Tama city, Japan			
				
			Estimated value	Standard error	Statistics	Probability	Estimated value	Standard error	Statistics	Probability
Capital	− >	Health status	1.00				1.00			
Capital	− >	Habitus	0.20	0.10	2.05	*	0.23	0.11	2.14	**
Capital	− >	Survival time	39.97	295.38	0.14	0.89	−69.98	138.15	−0.51	0.61
Habitus	− >	Health status	0.53	0.14	3.83	***	3.19	1.00	3.20	***
Habitus	− >	Survival time	−136.06	177.16	−0.77	0.44	−169.11	225.03	−0.75	0.45
Health status	− >	Survival time	470.73	238.21	1.98	*	127.57	44.48	2.87	**
Capital	− >	Cultural capital	1.00				1.00			
Capital	− >	Economic capital	0.42	0.09	4.48	***	0.99	0.22	4.45	***
Habitus	− >	Group interest	1.00				1.00			
Habitus	− >	Personal interest	0.63	0.13	4.91	***	6.04	2.33	2.59	**
Health status	− >	Social health	1.00				1.00			
Health status	− >	Physical health	0.53	0.07	8.09	***	0.49	0.04	11.95	***
Health status	− >	Mental health	0.23	0.07	3.56	***	0.28	0.05	5.31	***
Social health	− >	Spatial interaction	1.00				1.00			
Social health	− >	Human interaction	0.91	0.08	12.05	***	0.23	0.05	4.23	***
Mental health	− >	Self- evaluation	1.00				1.00			
Mental health	− >	Subjective feelings	3.01	0.80	3.77	***	1.97	0.33	5.99	***
Physical health	− >	Illness Type 1	−0.14	0.04	−3.90	***	−0.19	0.03	−5.98	***
Physical health	− >	Illness type 2	−0.14	0.04	−4.11	***	0.07	0.02	2.83	**
Physical health	− >	Discomfort symptoms	−0.17	0.04	−3.84	***	−0.14	0.04	−3.09	***
Physical health	− >	Living ability 1	1.00				1.00			
Physical health	− >	Living ability 2	1.02	0.05	22.66	***	0.93	0.04	23.22	***
Age	− >	Capital	−0.03	0.01	−6.76	***	−0.02	0.00	−5.29	***
Age	− >	Health status	0.00	0.01	0.66	0.51	−0.01	0.01	−1.86	0.06

*** < 0.001; ** < 0.01; * < 0.5.

### Generative structural relationships among capital, habitus, and healthy and their intensity changes in different fields

The standardized path coefficients of the models for Shenyang City shown in Figure 5 show that “capital” not only has a direct effect on “health status” (0.69) but also has an indirect effect on “health status” through “habitus” (the product of the path coefficients between capital → habitus → health status, i.e., 0.18 × 0.39 = 0.07). On the other hand, the direct effect of “capital” on “survival time” and the indirect effect of “survival time” through “habitus” did not pass the significance test, while having an indirect effect on “survival time” through “health status” (the product of the path coefficients capital → health → survival time, i.e., 0.69 × 0.34 = 0.24) as well as an indirect effect on “survival time” first through “habitus” and then through “health status” (the product of the path coefficients between capital → habitus → health → survival time, i.e., 0.18 × 0.39 × 0.34 = 0.03). At the same time, the standardized path coefficients of the models for Tama City shown in Figure 6 show the same patterns.

The above results validate Hypothesis 1, i.e., capital and habitus simultaneously affect health, in which capital dominates and also affects habitus—the three assuming a triangular generative structural relationship. The basic sources of health inequalities include (1) the direct effect of capital on health; (2) the indirect effect of capital on health through habitus, i.e., class habitus in a habitus that is partly dependent on capital has the class nature, and thus, is one of the sources of health inequalities.

**FIGURE 7:**
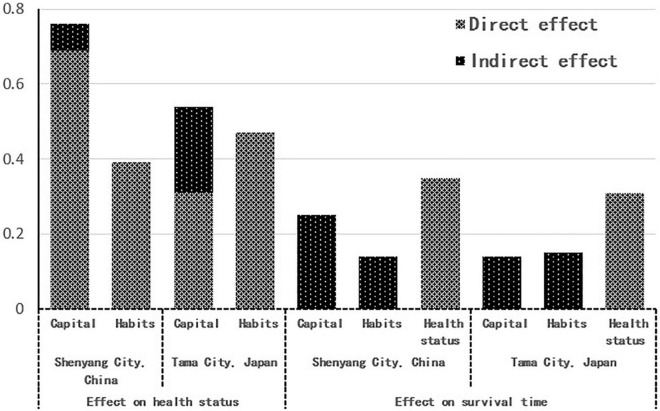
Direct and indirect effects of capital and habitus on health status and survival time.

The changes in the standardized path coefficients from the model of Shenyang City and to that of Tama City (Figures 5, 6) indicate that the direct effect of “capital” on “health status” significantly weakened (0.69 – > 0.31), but the direct effect of “habitus” on “health status” significantly strengthened (0.39– > 0.47). In particular, the direct effect of “capital” on “habitus” also significantly strengthened (0.18 – 0.49). At the same time, Figure 7 was generated based on the direct and indirect effects of “capital” and “habitus” on “health status” and “survival time” that are exported by the back end of AMOS, in which the sum of the direct and indirect effects is deemed as the total effect. The total effect of “capital” on “health status” and “survival time” significantly decreased from Shenyang City to Tama City (0.76– > 0.54 and 0.25– > 0.14, respectively), of which the direct effect of “capital” on “healthy status” markedly decreased (0.69– > 0.31), while the indirect effect of “capital” through “habitus” markedly increased (0.07– > 0.23).

The above results validate Hypothesis 2, i.e., from developing countries to developed countries, the macro-field conversion of a highly developed socio-economy and the highly sophisticated social welfare system, which leads to the weakening effect of capital on health in the micro-analysis, while that of habitus on health strengthens. In other words, the time-space conversion of the field not only changes the total amount and composition of the individual’s capital but also changes various macroscopic gravitational forces in the field.

### Difference in mechanisms of health inequalities in developing and developed countries

In the model of Shenyang City shown in Figure 5, the direct effect of “capital” on “health status” (0.69) was extremely strong, while the indirect effect of “capital” on “health status” and “survival time” through “habitus” (0.07 + 0.03) was very weak, indicating that the effect of “capital” on “health” mainly stems from a direct effect. These results validate Hypothesis 3, i.e., in the health field of developing countries, where socioeconomic development is still at an initial stage and the social welfare system is not yet sophisticated, the direct effect of capital on health is much greater than its indirect effect on health through habitus. The influence of class habitus that depends on capital on health is very weak, and the source of health inequalities is mainly the direct effect of capital.

In the model of Tama City shown in Figure 6, the direct effect of “capital” on “health status” (0.31) was weakened, and the indirect effect of “capital” on “health status” and “survival time” through “habitus” (0.23 + 0.14) strengthened, indicating that the effect of “capital” on “health” stems from both direct and indirect effects. These results validate Hypothesis 4, i.e., with a highly developed macro socio-economy and highly sophisticated social welfare system, changes in macro-field gravity reduce rather than eliminate health inequalities. The direct influence of capital on health is still present albeit diminishing. The indirect effect of capital on health through habitus strengthens, and class habitus has gradually become an important source of health inequalities in developed countries (Figure 7).

## Conclusion and discussion

### Main conclusion

In this study, we investigated the basic sources of health inequalities—those of health inequalities in developing countries and the new trend of the mechanism of health inequalities in developed countries to provide policy bases for countries at different development stages to cope with population aging and continuously reduced social inequality in the elderly. Using the same long-term fixed-cohort study method, we collected data on various controlled exposure factors, behavioral characteristics, and health status and survival time from 506 seniors in Shenyang City, China, and 569 seniors in Tama City, Japan, constructed generative structural equation models of capital, habitus, and health based on Bourdieu’s theory of social practice and the relevant correction conducted in this study, and compared and analyzed the health fields of China and Japan that represent developing countries and developed countries, respectively, to reveal different sources of health inequalities in the two countries. The conclusions of this study are as follows.

First, regarding the relationships of health with capital and habitus, capital and habitus simultaneously affect health. Moreover, capital is primary and also affects habitus, and capital, habitus, and health exhibit a triangular generative structural relationship.

Second, space-time conversion of field not only changes the total amount and structure of capital but, more importantly, changes various macro gravitational forces of the social environment; macro-field changes will bring about changes in the effects of capital and habitus on health practices at the micro-level.

Third, health inequalities are systemic health difference brought about by capital that reflects social structural factors, with two main sources: one is the direct effect of capital on health, and the other is the indirect effect of capital through its influence on habitus, i.e., class habitus that is controlled by capital has class attributes, which is also one of the sources of health inequalities.

Fourth, in the health fields of developing countries where socioeconomic development is still at an initial stage and the social welfare system is not yet sophisticated, macro-social structures differ significantly, and the direct impact of the individual’s capital on health is much greater than that of habitus, while the effect of capital through class habitus on health is very weak; health inequalities are mainly from the direct impact of capital.

Fifth, in the health field of developed countries where the macro-society is highly developed and the social welfare system is highly sophisticated, the direct effect of capital on health is still present but is relatively diminished, while the indirect effect of capital on health through class habitus is increased, health inequalities are overall reduced rather than eliminated, and the source of health inequalities has gradually shifted from capital’s direct effect to class habitus that is dependent on capital.

### Discussion of sources of health inequalities

Developing countries are in the initial stages of socioeconomic development. Their social welfare systems have yet to be improved. The material living environment and medical resources that are decisive in sustaining life are generally deficient. Wide gaps in resource levels are present among all different social strata, and only groups with high capital for individuals can gain access to abundant food, timely medical care, and a good living environment. Dahlgren and Whitehead (2006) summarized the social structural causes of health inequalities from five aspects: (1) differences in power and resources, (2) differences in exposure to health risk factors, (3) differences in damage given the same exposure, (4) differences in life experience, and (5) differences in the social and economic consequences of adverse life events. This theoretical explanation emphasizes the role of the first subsidiary character of capital, i.e., the direct effect of its role in obtaining material resources on disease prevention, which can well explain the source of health inequalities under the background of the early stage of socioeconomic development when the income disparity is high and the social security system is unsophisticated. The social security system of China, which is still a developing country, is not sophisticated, and for various insurance types, e.g., the basic medical insurance for urban workers, basic medical insurance for urban residents, the new rural cooperative medical care, public health, and commercial insurance, insurance premiums, and benefits for policyholders vary tremendously. Behind the difference in social security systems is the difference in the macro-social structures, causing the subsidiary material resource attribute of capital to naturally become the main source of health inequalities, while the effect of class habitus is indirectly affected by capital on health is very weak.

Japan, as a developed country, has diverse medical insurance systems such as health insurance, mutual aid, national health insurance, and late-stage medical insurance for seniors. Different medical insurances charge different premiums but provide more or less similar benefits; among them, the proportions of payments made by seniors decrease with age, and out-of-pocket portions can be reimbursed when the medical bill exceeds a certain amount, especially after 2000, when the elderly long-term care insurance system that benefits the entire population was implemented (Zhao et al., 2014, p. 80–123). In the context of altered external macro-social structures, the direct effect of the individual’s capital on health has changed, i.e., the direct effect of the material resource attribute of capital has weakened, which to some extent reduces capital-derived health inequalities.

However, the so-called health phenomenon includes not only various disease types, e.g., famine, malnutrition, and infectious diseases, that are frequent in developing countries but also systematic differences in populations with various health phenomena such as lifestyle-related diseases, psychological disorders, and social hazards. Therefore, it is very difficult for the material resources attribute of capital to explain the health inequalities in a health field with a highly developed socio-economy and highly sophisticated social welfare system. This study revealed that the direct impact of the capital of older Japanese people on health is gradually weakening; on the contrary, the influence of social capital on health through habitus in pursuit of life quality is slowly strengthening. Material resources have a decisive role in health but also have limitations; with improvements in nutritional status or medical level, both health status and life expectancy can be improved; however, after reaching the level that meets bodily needs, the relationship between the two will be weakening or even disappearing. In the case that the society as a whole reaches the nutrition and medical care level that meets bodily needs through macro-social welfare improvements, the influence of differences in the consciousness, ability, and opportunity of pursuing quality of life on health has begun to show, i.e., the influence of class habitus is gradually increasing. Therefore, the highly developed socio-economy and the highly sophisticated social welfare system have not completely eliminated health inequalities but only altered the ways in which health inequalities arise, i.e., shifting from the direct effect of capital to class habitus that is indirectly affected by capital. After the time-space conversion of the health field, the mechanism of health inequalities has changed, and the influence of material resources, which are the first subsidiary attribute of capital, on health differences still exists but is gradually declining, while the influence of the pursuit of quality of life, which is the second subsidiary attribute of capital, on health differences is gradually rising.

Wang (2012) showed that like major developed countries such as European countries and the United States, China also exhibits significant health inequalities such that the likelihood that an individual possesses and maintains a healthy lifestyle, which directly affects the health of the individual, increases with the individual’s socioeconomic status. The hypothesis of the above study is similar to ours, but they have different theoretical perspectives and analytical methods, which lead to different interpretations of the sources of health inequalities. First, health in the above-mentioned study refers to a single variable of subjective perception of health obtained through cross-sectional surveys, whereas that in this study refers to the health statuses and survival times obtained through cross-sectional and follow-up surveys. Second, the effect of socioeconomic status on lifestyle and that of lifestyle on health hypothesized in the above-mentioned study were verified separately through two statistical models, whereas in this study, the indirect and direct effects of capital were simultaneously validated in the same structural equation model. Third, the lifestyle as understood in the above-mentioned study is a medically reasonable instrumental means, e.g., walking, jogging, and a rigid daily schedule, and in general, for seniors, these passive daily activities can be readily accomplished but fail to explain the large health differences among the seniors; the lifestyle perceived in this study is the habitus of interest in the pursuit of the meaning of life such as personal entertainment and group communication activities, with plenty of fun and cultural significance in the behavior itself. Fourth, the above-mentioned study argued that the mechanism of health inequalities in China as a developing country is similar to that in developed countries such as the European countries and the United States, whereas the present study revealed that the sources of health inequalities in health fields with different macroscopic gravitational forces differ. At present, China is still at the initial stage of socioeconomic development, and health inequalities mainly stem from the direct effects of capital, i.e., the differentiation in medical resources and food resources are the main sources of health inequalities. However, in developed countries, the highly developed socio-economics and highly sophisticated social welfare systems contribute to the reductions in health inequalities, and the source of health inequalities has begun to shift from the direct effect of capital to its indirect effect, i.e., class habitus will become an important source of health inequalities.

### Discussion of Bourdieu’s theory of social practice

Swartz (2008) argued that although Bourdieu’s sociological theory has been increasingly put into use in the field of sociology, it is still difficult to apply the three core concepts, i.e., habitus, capital, and field, to a study; moreover, the three concepts have rarely been addressed from the relationship perspective that is crucial to Bourdieu’s theory. In this study, on the theoretical bases of the linear generative relationships among Bourdieu’s three concepts, i.e., capital, habitus, and practice, and the subconscious guidance of habitus on practice under the constraints of capital, we postulated that capital precedes habitus in the control over practice, i.e., direct effect, and tested the capital-based triangular generative structure relationships among capital, habitus, and practice, which represents a new exploration of Bourdieu’s generative relationship theory. Moreover, based on the theory that Bourdieu’s space-time field conversion refers to the temporal changes of the total amount of capital or that the composition and proportion of capital lead to class track changes, in this study, we proposed that a more important space-time field conversion is the change that is external to the individual macroscopic field gravitational forces, i.e., under a highly developed macroscopic socio-economy and highly sophisticated social welfare system, the effects of the individual’s capital on habitus and practice have changed from a microscopic perspective. This expansion and upgrade in analysis perspective not only achieve the application of the three core concepts (capital, habitus, and field) in the same study but also validates the importance of relationships emphasized by Bourdieu’s theory, rather than the isolated effect of total effect; in particular, the new interpretation of space-time field conversion also achieved the unity of micro-analysis and macro-analysis. At the same time, according to Bourdieu, social inequality structures are based on the objective social structures derived from the uneven distribution of different types of social capital, based on which, we proposed that basic resources such as food and medical resources are essential for sustaining life can achieve gradual equalization through macro-social development and the adjustment of macro-social institutions, which has changed the authority of the individual’s capital in obtaining material resources; overall, changes in macro-social structures have the effect of reducing social inequality.

Anheier et al. (1995) distinguished the different roles of various types of capital in detail and argued that a social structure and its different positions can be determined based on the type and amount of capital accumulation; moreover, the significant differences in cultural capital and social capital distinguish elite jobs from non-elite ones, and relative to cultural and social capitals, economic capital plays a less important role in understanding the social structures of the cultural sphere. Although models of this study can also distinguish different functions of various types of capital, we posit that a latent common factor extracted from various types of capital has greater explanatory significance when discussing the social structures of overall inequalities. The capital with the social structure positional attribute should not have any association with health, but its two subsidiary attributes, i.e., obtaining material resources and pursuing quality of life, are related to human health. Material resources, which are a direct influencing factor of capital on health, are indispensable basic conditions for maintaining life and can reduce various unfavorable factors such as diseases, having the effect of avoiding negative health factors. Quality of life, which is an indirect influence factor of capital on health, is an active pursuit of the meaning of life and can stimulate the production of various favorable factors that increase body energy, having the effect of increasing positive health factors.

Emirbayer and Victoria (2008) argued that many studies have almost completely ignored habitus, the third major concept of Bourdieu’s theory; the absence of the concept of habitus will make the concepts of both field and capital meaningless. This further confirms the misuse of Bourdieu’s viewpoints and the lack of understanding of their potential usefulness. Moreover, Bourdieu argued that habitus has similarities, which lead people to believe augments such as that their thoughts and deeds generate favorable or unfavorable impressions in others offering a thorough explanation that habitus has class attributes (Bourdieu, 2017, p. 4–21). In this study, we focused on the concept of habitus and believe that habitus overall reflects the behavioral aspiration and interest of the individual’s subjective initiative features, parts of which, however, depend on capital to realize, and can be decomposed into personal habitus and class habitus. Thus, in this study, we proposed a new source of health inequalities, i.e., systematic health differences caused by class habitus that is controlled by capital, which is especially more profound in comparison to the health fields of developing and developed countries. Therefore, this conclusion also verifies the view of Francois (2009) that the greater the change in a social environment, the more significant the effect of analyzing the actor’s behavior when using habitus as a tool.

Lastly, the results of this study can provide theoretical bases for developing and developed countries to formulate policies to reduce or eliminate health inequalities. In the initial stage of social development and social welfare system improvement, national policy and social intervention are still focused on improving the material life and medical resources for the elderly, and with a highly developed socio-economy and highly advanced social welfare system, the focus of national policies and social intervention should be gradually shifted to improve the autonomy and the aspirational aspects of the elderly to assist them to realize their self-value. However, despite the above-mentioned innovative results obtained in this study, there are certain limitations that cannot be resolved in a single study. A comparative study of China and Japan, which represents developing and developed countries, respectively, requires further discussions of the generality and particularity of social development in both countries. Second, limited by the commonality of the survey method used in Shenyang City and Tama City, only two operational variables were assigned to some latent concepts, without entirely consistent follow-up times, which needs further methodological coordination to facilitate international comparative studies. Third, this study was intended to investigate the mechanism of health inequalities among the elderly, and the exploration of Bourdieu’s theory of social practice needs further discussion at the theoretical level.

## Data availability statement

The original contributions presented in the study are available upon reasonable request, further inquiries can be directed to the corresponding author/s.

## Ethics statement

Ethical review and approval was not required for the study on human participants in accordance with the local legislation and institutional requirements. Written informed consent from the patients/participants or patients/participants legal guardian/next of kin was not required to participate in this study in accordance with the national legislation and the institutional requirements.

## Author contributions

Both authors listed have made a substantial, direct, and intellectual contribution to the work, and approved it for publication.
